# The Landscape of AhR Regulators and Coregulators to Fine-Tune AhR Functions

**DOI:** 10.3390/ijms22020757

**Published:** 2021-01-13

**Authors:** Marco Gargaro, Giulia Scalisi, Giorgia Manni, Giada Mondanelli, Ursula Grohmann, Francesca Fallarino

**Affiliations:** Department of Medicine and Surgery, University of Perugia, 06132 Perugia, Italy; marco.gargaro@unipg.it (M.G.); giulia.scalisi@gmail.com (G.S.); manni.giorgia87@gmail.com (G.M.); giada.mondanelli@unipg.it (G.M.)

**Keywords:** arylhydrocarbon receptor (AhR), transcription factors, coregulators, nuclear coactivator, immune regulation

## Abstract

The aryl-hydrocarbon receptor (AhR) is a ligand-activated transcription factor that mediates numerous cellular responses. Originally investigated in toxicology because of its ability to bind environmental contaminants, AhR has attracted enormous attention in the field of immunology in the last 20 years. In addition, the discovery of endogenous and plant-derived ligands points to AhR also having a crucial role in normal cell physiology. Thus, AhR is emerging as a promiscuous receptor that can mediate either toxic or physiologic effects upon sensing multiple exogenous and endogenous molecules. Within this scenario, several factors appear to contribute to the outcome of gene transcriptional regulation by AhR, including the nature of the ligand as such and its further metabolism by AhR-induced enzymes, the local tissue microenvironment, and the presence of coregulators or specific transcription factors in the cell. Here, we review the current knowledge on the array of transcription factors and coregulators that, by interacting with AhR, tune its transcriptional activity in response to endogenous and exogenous ligands.

## 1. Introduction

The aryl-hydrocarbon receptor (AhR) is a ligand-activated transcription factor that can recognize a large variety of molecules of both endogenous and exogenous origin. Because of this property, AhR acts as a crucial sensor of the cellular presence of a myriad of compounds capable of crossing the cell membrane [1,2]. The list of AhR ligands is impressive and striking in its diversity. Due to this ligand’s promiscuity and consequent pleiotropic functions, AhR plays critical roles in several physiologic processes such as xenobiotic metabolism, immune responses, cell proliferation, differentiation, and apoptosis [3,4]. Moreover, AhR involvement has been reported in the development and maintenance of several pathological conditions [3].

The different outcomes of AhR activation may depend on the specific ligand, the environment (i.e., conditions which can lead to the concomitant activation of other cellular pathways), and the interaction with specific comodulators of gene transcription and/or other transcription factors as well [5]. Thanks to several studies [6,7,8,9,10,11], the role of AhR has particularly emerged in the cells of the immune system, in which the receptor acts to balance the effector and regulatory arms of responses and thus represents an attractive target for future strategies of therapeutic immune modulation. Here we review the numerous partners able to interact with AhR, potentially contributing to cell-specific AhR functions and fine-tuning immune responses.

## 2. AhR, a Promiscuous Ligand-Activated Transcription Factor Regulating Immune Responses

### 2.1. Canonical AhR Activation by Endogenous and Exogenous Ligands

**Figure 1:**
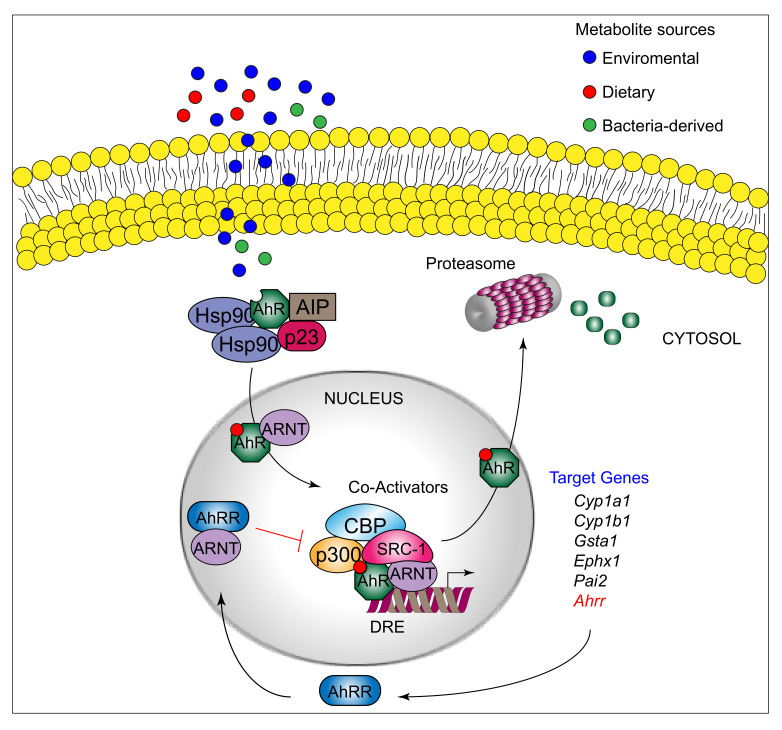
The canonical aryl-hydrocarbon receptor (AhR) pathway. AhR ligands from different sources can cross the plasma membrane and bind to AhR. This allows the translocation of the ligand–receptor complex to the nucleus. In the nucleus, AhR heterodimerizes with its partner ARNT (AhR Nuclear Translocator, also called HIF-1β). The heterodimer binds specific DNA regions located in the promoter of target genes, named dioxin response elements (DRE). AhR-ARNT induces the transcription of target genes, including members of the *Cyp1a* and *Cyp1b* families, by the recruitment of various coactivators, such as CBP/p300 (cAMP response element-binding protein) and SRC-1 (steroid receptor coactivator 1). AhRR further suppresses AhR transcriptional activity. Then, after being exported out of the nucleus, AhR is rapidly degraded in the cytoplasmic compartment by the proteasome.

AhR can be activated by a structurally diverse spectrum of synthetic and environmental chemicals, including dietary components, microbiota-derived factors, and endogenous tryptophan (Trp) metabolites. The prototypical AhR exogenous ligand is represented by 2,3,7,8-tetrachlorodibenzo-p-dioxin (TCDD), an environmental contaminant whose signaling has been largely studied. Halogenated dioxins, polycyclic aromatic hydrocarbons, and halogenated biphenyls represent the best characterized, high affinity planar aromatic and hydrophobic ligands of AhR. For a long time, it was believed that only molecules with specific physiochemical characteristics, such as hydrophobic structures with planar shape, could interact with AhR [12]. More recent research has shown that this “dogma” is not entirely correct. Indeed, a large number of structurally diverse natural, endogenous, and synthetic AhR ligands, having little similarity with prototypical AhR ligands, can activate the receptor [13]. Moreover, the discovery of these novel compounds able to interact with AhR, many of which have an endogenous source, has prompted more interest in the study of the major physiological roles of AhR. At present, the more deeply studied AhR endogenous ligands are those derived from Trp. The three major Trp derivatives that appear to contribute to the outcome of gene transcriptional regulation by AhR include kynurenines [14] in mouse lymphoid tissue dendritic cell (DCs), 6-formylindolo[3,2-b]carbazole (FICZ) in human skin keratinocytes [15], and microbiota-derived indole-3-aldehyde (IAld) in innate lymphoid cells of the gut in mice [16]. Although characterized by different physiochemical characteristics, these ligands are able to interact with AhR within the ligand binding domain, thus causing a conformational change translating into AhR transcriptional activity (Figure 1).

AhR belongs to the bHLH-PAS family of structurally related proteins, as it contains a basic Helix–Loop–Helix motif in the *N*-terminus and a PER/ARNT/SIM-homology domain in the *C*-terminal adjacent region. The PAS domain contains two imperfect repeats of 50 amino acids, namely, PAS A and PAS B, and is thought to serve as an interactive surface for dimer formation; in particular, PAS B represents the ligand-binding domain. The *C*-terminal half amino acidic sequence mediates transactivation activity through several specific sites, for instance a glutamine-rich box [17]. More specifically, the transactivation domain (TAD) facilitates the dynamic interplay between AhR and coactivators or coregulators [18,19]. In its inactive form, AhR is sequestered in the cytosol as part of a large complex containing several proteins, including the molecular chaperone HSP90 [20], the cochaperone p23 [21], the protooncogene SRC (c-SRC) tyrosine protein kinase [22], and the hepatitis B virus X-activating protein 2 (XAP2) [23] (also known as AhR-interacting protein or ARA9) [24,25]. The interaction of mammalian AhR with selected ligands induces the detachment of the receptor from this complex, leading to AhR nuclear translocation and promotion of AhR transcriptional activity [26] Specifically, the interaction of AhR with an agonist will induce conformational changes that trigger the receptor to dissociate from the chaperon complex, an event that drives AhR binding to the AhR nuclear translocator protein (ARNT) in the nucleus. However, evidence demonstrating that AhR may not necessarily dissociate from HSP90 to translocate to the nucleus has also been provided in human cells [27] In the nucleus, the AhR/ARNT complex interacts with multiple consensus sequences in the promoter of several genes, designated as xenobiotic responsive elements (XREs; also AhRREs and dioxin responsive elements, DREs). These events require ATP-dependent chromatin remodeling factors (e.g., BRG-1) and the recruitment of coactivators, such as the CBP/p300 histone-acetyltransferases by the ARNT moiety, and RIP140 and SRC-1 by the AhR fraction, which transmit the transactivation activities of the heterodimer [28]. In addition, other DNA elements cis-acting with XREs allow the AhR/ARNT complex to operate in synergy with other transcription factors. This control of transcriptional activity is known as the “canonical” genomic pathway of AhR signaling [29]. Different AhR ligands in specific cell compartments may trigger the formation of specific AhR protein complexes, which differ in their composition, leading to differential gene expression [30]. In addition to the genomic functions, several studies have shown that AhR regulates cellular responses through ARNT-independent, nongenomic mechanisms that involve Ca^2+^, c-SRC, cytosolic phospholipase A2 (cPLA2), and COX-2. In particular, after human and mouse AhR activation, c-SRC is released from the AhR/HSP90/c-SRC complex and thus acquires the ability to phosphorylate multiple cellular targets [31,32]. Moreover, it has been reported that AhR can act as a ligand-dependent E3 ubiquitin ligase, thus leading to the degradation of AhR-interacting proteins through the proteasome [33].

### 2.2. AhR Expression and Functions in Immune Cells: A Territory for Exploring the Intricacies of AhR Interaction with Coregulators?

AhR was originally identified in mouse cells and next identified also in human tissues, where the highest expression was found to be similar to mouse cells in the lung, thymus, kidney, and liver [34]. The elevated expression levels of AhR in barrier organs such as the skin, gut, and lung and the ability of AhR to bind many exogenous and endogenous compounds have recently attracted the attention of immunologists [7,10,35]. Although AhR-deficient mice do not exhibit a specific phenotype in steady state conditions, studies over the last 20 years have revealed that exposure of AhR deficient mice to inflammatory stimuli determines substantial immune defects in the body’s main barrier sites, such as skin [36,37], the lungs [38,39], and the gastrointestinal tract [40].

This evidence highlights the role of AhR as a gatekeeper of both physical and immunological barriers. The crucial importance of the AhR pathway in the regulation of the intestinal immune system has been demonstrated by genome-wide association studies that have identified the *AHR* gene as a susceptibility locus in inflammatory bowel diseases [41]. In fact, ablating AhR in CD11c^+^ cells (i.e., mostly represented by DCs) perturbs intestinal epithelium development and intestinal immunity [42]. Moreover, AhR plays an essential role in murine RORγt(+) ILC3, (type 3 innate lymphoid cells) maintenance and function, cells which are essential for gut immunity presumably through the production of interleukin-22 (IL-22) [40]. A recent study unveiled that AhR expression in epithelial intestinal cells is responsible for the metabolic clearance and detoxification of AhR ligands via induction of P4501 (CYP1) enzymes and that this negative feedback mechanism protects from excessive AhR activation [43]. Therefore, an AhR-mediated circuitry appears to be mandatory for the development and immunologic homeostasis of the gut.

Earlier studies demonstrated the crucial role of AhR in adaptive immune responses for both T(reg) and T(H)17 cell differentiation in a ligand-specific fashion, pointing to a unique target for therapeutic immunomodulation [44]. Intriguingly, it is now emerging that the AhR expression profile can change during the course of differentiation and activation in many immune cells. In this regard, a typical example is constituted by T cell subsets. In fact, AhR is more expressed in mouse T helper type 17 (Th17) cells compared to non-polarized activated Th0 cells as well as to Th1, Th2, and regulatory T (Treg) cells [45]. Moreover, numerous studies have reported the expression of AhR in several other immune cell types including B cells, mucosal cells, and antigen-presenting cells (APCs) [10].

With respect to AhR ligands, the controversial effects of AhR agonists have been observed in the control of T cell function, especially in autoimmune diseases. For example, TCDD treatment of CD4^+^ T cells results in an AhR-dependent induction of Foxp3^+^ Treg cells in vitro, and TCDD administration in mice with experimental autoimmune encephalomyelitis (EAE; a model of multiple sclerosis) results in Treg cell expansion and suppression of the disease [44]. In contrast, Veldhoen et al. demonstrated that FICZ, locally administered during EAE, enhances the differentiation of Th17 cells [45], while in other studies the systemic administration of FICZ significantly reduces the signs of EAE [46]. These data strongly highlight the role of tissue microenvironment in conditioning the outcome of AhR activation in specific immune cells.

Several cells have been reported to express AhR, mostly correlating with immunoregulatory phenotypes. Specifically, a crucial role of AhR has been reported for human type 1 regulatory T cells (Tr1), in which AhR, in concert with c-Maf, controls IL-21 and IL-10 production [47]. Different levels of AhR are also expressed in different subsets of mouse and human B lymphocytes [48,49,50]. In 1990, Kerkvliet et al. reported that AhR activation by environmental toxins can be responsible for the suppression of humoral immune responses [51]. More recent studies demonstrated that B-cell receptor activation leads to AhR induction in B cells, and that AhR-deficient B cells proliferate less well than their wild-type counterparts [52]. Microarray analysis of myeloid cells indicated AhR expression also in both macrophages and DCs [35], which was also confirmed by data on AhR protein expression both in mouse and human counterparts [14,53,54,55]. The levels of AhR expression increase upon LPS and other Toll-like receptor (TLR) ligand engagement in macrophages and DCs and B cells [32,56,57,58]. Specifically, recent studies demonstrated that AhR regulates the differentiation and functions of CD19^+^CD21^hi^CD24^hi^ regulatory B (Breg) cells, producing IL-10 and thus limiting the differentiation of inflammatory B cells [57]. Interestingly, AhR-deficient APCs also show a defect in IL-10 production [59].

The role of AhR in regulating DC functions has been extensively studied. At present, it is known that AhR activation alters DC differentiation and innate functions. Bone marrow-derived DCs (BMDCs) from C57BL/6 mice, generated in the presence of TCDD, decrease CD11c expression and increase MHC class II and CD86 expressions [60]. Similar effects were shown for other AhR ligands, such as indole-3-carbinol (I3C) and indirubin-3′-oxime (IO). Both I3C and IO decrease the expression of CD11c, CD40, and CD54, while increasing the expression of MHC class II and CD80. Moreover, following lipopolysaccharide (LPS) activation, I3C and IO suppress the production of pro-inflammatory mediators, including TNF-α, IL-6, IL-12, and nitric oxide but increase IL-10 levels. Additionally, immunoregulatory genes, such as those coding for aldehyde dehydrogenase (ALDH1A), indoleamine 2,3-dioxygenase 1 (IDO1), and TGF-β are up-regulated after treatment with I3C or IO [61]. Moreover, it was also demonstrated that activation of AhR in DCs is able to induce SOCS2 expression, thus limiting the production of pro-inflammatory IL-6 and IL-12 [62]. Concomitantly, DC activation by the AhR ligands TCDD and the tryptophan metabolite kynurenine promotes the expression of IDO1 and IDO2 immunoregulatory enzymes [14] which, in turn, by catalyzing further production of kynurenine, sustain the induction of TGF-β and lead to the acquisition of tolerogenic properties by the DCs [32,59].

Overall, although AhR is expressed in several immune cell types, the outcome of AhR activation by specific ligands may be different. This difference may be ascribed to the presence of AhR partners other than prototypical ARNT in different cell types.

## 3. Transcription Factor Families Binding and Regulating AhR Functions

Canonical AhR signaling, described more than 50 years ago, has been extensively studied in the field of toxicology. In recent years, it has received high levels of attention in the field of immunology, as described in detail in several excellent reviews [9]. Compelling evidence has reported that AhR can also alter gene transcription through sites distinct from the canonical consensus XRE. For instance, the ligand-activated mouse and human AhR/ARNT dimer can directly interact with the unliganded estrogen receptor, promoting the formation of a transcriptionally active complex capable of binding estrogen response elements [63]. Moreover, mouse AhR can activate alternative signaling routes by its specific interaction with molecules of other intracellular pathways, such as signal transducers and activator of transcription (STAT) family members [54], thus resulting in regulatory functions. Additional evidence is accumulating for direct AhR–DNA binding in conjunction with the NF-κB transcription factor in mouse B cells and macrophages, leading to the regulation of the NF-κB signaling pathway and gene transcription [64].

### 3.1. ARNT/HIF1α

The cellular response to hypoxic conditions involves the activation of hypoxia-inducible factors (HIFs), a family of DNA-binding proteins that induce the transcription of numerous genes involved in metabolic adaptation, angiogenesis, glycolysis, and cell survival [65]. HIF1α is a basic Helix–Loop–Helix–PAS heterodimer regulated by cellular O_2_ tension [66]. The canonical AhR pathway involves the interaction of AhR with the specific partner ARNT, also called HIF1β, thus promoting AhR–DNA binding at the XRE sites. ARNT also binds to the hypoxia inducible factor 1 (HIF1α) leading to binding to hypoxia response elements [67]. Thus, since ARNT can be both used by AhR and HIF1α, this can result in a competition between AhR and HIF1α for heterodimerization with ARNT. Specifically, the ARNT/ HIF1α complex can bind specific DNA cognate sequences, enhancing the expression of a series of hypoxia-dependent genes and indirectly leading to suppression of AhR transcriptional activity in rat cells [68]. Similarly, in another study, aerobic glycolysis supports Tr1 cell differentiation through a metabolic program controlled by HIF1-α that antagonizes AhR activity during human and mouse Tr1 cell differentiation [69].

### 3.2. AhR/RB

Retinoblastoma (RB) is a tumor suppressor protein involved in cell cycle regulation. One of its functions consists of repressing the expression of E2F target genes, such as enzymes involved in nucleotide and protein synthesis, which are essential for progressing from the G1 to S phase. RB binds E2Fs, either masking transactivation domains or reinforcing gene repression [70,71]. Therefore, RB prevents DNA replication and blocks the cell cycle in the G1-phase.

Long-term evidence has shown that TCDD inhibits cell proliferation [72,73]. One contributing mechanism relies in the direct interaction between AhR and hypophosphorylated RB. RB was reported to coimmunoprecipitate with ligand-bound AhR/ARNT, and yeast-two hybrid assays indicated that the interaction occurs with the AhR moiety but not with ARNT [74]. The AhR RB-binding region was identified in the *N*-terminal half of the protein sequence, within the bHLH region or an LXCXE motif in the PAS B domain, assisted by the glutamine-rich box at the *C*-terminal transactivation domain. Via interaction with RB, AhR strengthens repression of E2F-dependent gene expression, likely protecting RB from CDK2-mediated phosphorylation or by inducing genes encoding CDK inhibitory proteins, such as p27kip1. Thus, in the presence of specific ligands and through this interaction, AhR slows down cell cycle progression in rat and human studies [74,75,76].

### 3.3. AhR/E2F1

AhR can also directly interact with E2F1, which transactivates genes essential not only for cell cycle progression, but also for promoting apoptosis. In particular, cellular stress by DNA damage results in phosphorylation of E2F1 by checkpoint kinase-2 (CHK2). This leads to the stabilization of the transcription factor on its DNA cognate sequence and induction of pro-apoptotic genes such as Apaf1 and p73 [77]. Whether the formation of the AhR/E2F1 complex coactivates or represses E2F1 target genes transcription is still unclear, but it may likely depend on which cells and tissues are involved [78].

It has been observed that AhR-null mouse embryo fibroblasts are more susceptible to oxidative stress, DNA damage, and E2F1-controlled apoptosis than wild type cells [77]. However, silencing the E2F1-coding gene in these cells by small interfering RNA (siRNA) slows down cell death. Notably, coimmunoprecipitation experiments demonstrated that, upon treatment with TCDD, AhR and E2F1 interact in RB-deficient cells. Chromatin immunoprecipitation assays revealed that the complex is recruited at the 5′-flanking regions of Apaf1 and p73, which contain E2F1 consensus sequences but not AhREs, thus repressing gene induction and inhibiting apoptosis. Human AhR overexpression in another cell model led to the same outcome [77]. These studies showed that the AhR-E2F1 interaction adds to the effects induced by the AhR/RB complex in suppressing E2F1 activity [77]. Another study, however, reported that AhR overexpression or ligand activation enhances human lung cancer A459 cell proliferation. Moreover, exposure to the AhR ligand 3-MC increases E2F1-mediated transcription in an AhR-dependent manner, via the formation of an AhR/ARNT/E2F1 complex binding the E2F1 consensus sequence [78].

### 3.4. AhR/ERα and AhR/AR

The estrogen receptor α (ERα) and the androgen receptor (AR) belong to the nuclear receptor family of ligand-activated transcription factors and mediate signals from female and male sex hormones, respectively.

A number of studies have reported on the anti-estrogenic effects of numerous exogenous AhR ligands. Upon treatment with the toxic molecule 3-methylcolantrene (3-MC), human AhR can interact with ERα even in the absence of 17β-estradiol, and acts as a coactivator of the estrogen responsive elements (EREs), thus promoting the transcription of ERα target genes [79]. Interestingly, polycyclic aromatic hydrocarbons can be metabolized into ERα agonists and, in particular, 3-MC, which has been observed to directly bind the ERα nuclear receptor [63,80]. Conversely, in MCF-7 breast cancer cells exposed to TCDD, ligand-bound AhR recruits ERα to the XREs located upstream of the *CYP1A1* and *CYP1B1* genes. Moreover, cotreatment with estrogens seemed to potentiate this process [79]. It is not well-defined yet whether the two factors are directly physically interacting or require the involvement of additional protein mediators. Moreover, it is still unclear whether such collaboration may result in the increased or reduced inducibility of CYP1 enzymes [81,82]. Notably, these effects might also be dependent on the specific cell type where the interaction occurs. An enhanced expression of CYP1 isoenzymes, which are involved in steroid hormone metabolism [83], would entail a sort of negative feedback loop modulating ERα activity. This specific aspect is consistent with the AhR-mediated downregulation of estrogenic signals. In particular, the agonist-activated AhR/ARNT heterodimer directly associates with ER-α and ER-β [63]. This association resulted in the recruitment of unliganded ER and the coactivator p300 to estrogen-responsive gene promoters, leading to attenuation of ER-mediated estrogenic effects.

Moreover, additional mechanisms involving AhR can also be postulated. In particular, AhR may favor ERα ubiquitination [84], while the AhR/ARNT dimer may suppress ERE-controlled transcription by binding to XREs [85]. The mechanism by which ER-mediated estrogen signaling is modulated by a coregulatory-like function such as AhR/ARNT remains incompletely understood. Potential mechanisms involve the modulation of ER signaling by direct AhR/ARNT heterodimer interactions suppressing ER mediated gene expression, or by steric hindrance of ER DNA binding due to overlapping or proximity of XRE and ERE sites [63,86].

Controversial results have been obtained for the functional relationship between AhR and AR. Activation of AhR by exogenous ligands has been reported to antagonize AR signaling. For example, TCDD can block the AR-dependent proliferation of prostate cancer cells [87]. The interference with androgenic signaling has been ascribed to AhR’s ability to promote the proteasomal degradation of AR [33]. However, these observations do not explain the carcinogenic effects of AhR. The use of a constitutively active AhR construct lacking the ligand binding domain has in fact revealed that, besides promoting AR proteasomal degradation, AhR can act as a transcriptional coregulator for the unliganded AR [88] and thus promote androgenic signaling in human cells.

### 3.5. AhR/RelA and RelB

It has long been known that inflammatory stimuli suppress CYP450 hepatic enzyme expression, suggesting that, among inflammatory pathways, canonical NF-κB may regulate AhR activity. Indeed, exposure to TNFα and LPS decreases TCDD-induced *CYP1A1* gene expression through RelA activation [89]. Intriguingly, RelA and AhR interplay can result in either suppression of or cooperation with each other’s activity [90]. Furthermore, this interplay may occur via physical interaction between the two transcription factors. Such a complex was first observed in an Hepa1 cell line upon TCDD treatment, along with the mutual suppression of AhR and NF-κB activities [91]. The main AhR partner, ARNT, was not found to be associated with RelA, suggesting that AhR-binding to RelA does not necessarily take place in the nucleus. In addition, the existence of the RelA/AhR complex was hypothesized in a DC line, leading to downregulation of TNFα– and anti-CD40–stimulated signals [58]. Further, in vitro experiments excluded direct interference with AhR’s ability to bind XREs. However, specific effects on selected steps of gene transactivation, such as chromatin remodeling and transcription elongation in the presence of TNF, were suggested [92]. Another study in an MCF-7 breast cancer cell line supported RelA involvement in repressing *CYP1A1* expression upon treatment with the proinflammatory cytokine IL-1β [93]. More recently, activation of AhR by TCDD and β-naphtoflavone (β-NF) was found to decrease the protein levels of the pro-inflammatory cytokines, TNF-α, IL-6, and IL-12, in mouse macrophages activated by LPS and IFNγ, an event mediated by AhR-dependent RelA/p65 ubiquitination and proteasomal and lysosomal degradation [94].

RelB is another member of the NF-κB family of transcription factors, structurally and functionally different from the other two transcriptionally active members, i.e., RelA and c-Rel [95,96]. It has been earlier reported that, when mice are treated with LPS or cigarette smoke, AhR deficiency heightens the inflammatory response in the lungs via the recruitment of neutrophils and secretion of IL-6, TNFα, and via keratinocyte chemoattractant (KC), the murine human IL-8 homolog. This finding correlated with an accelerated loss of nuclear RelB protein levels, suggesting that AhR, at least in mouse studies, may help in maintaining the RelB expression needed to downregulate RelA-mediated inflammatory responses [97]. The precise nature of the DNA binding site remains unresolved. In fact, it is still unclear whether a RelA driven interaction with a NFκB response element or binding to a novel AhR/RelA response element may occur. Other studies also reported that AhR activation by TCDD increases IL-8 expression in vitro by a mechanism involving AhR and RelB heterodimerization, forming a complex at a ‘XRE-like’ motif (5′-GGGTGCAT-3′) in the IL-8 promoter in mouse and human settings [98].

Both AhR and RelB are known to play a role in DC differentiation and function [60]. Notably, it has been reported that TCDD promotes RelB nuclear translocation in wild type DCs but not in AhR-null ones [99]. Specific genes found to be regulated in these settings were those coding for IDO1 and IL-10, leading to the induction of tolerogenic DCs [99]. Accordingly, recent studies provided evidence for the important role of RelB in the transcriptional regulation of cytokines, such as IL-6 and IL-22, and enzymes such as IDO1 in bone marrow-derived macrophages stimulated by selected AhR ligands [100]. Specifically, TCDD and FICZ increased those cytokines whereas I3C significantly suppressed them.

Overall, evidence has been accumulating to indicate that there are close interactions between AhR and NF-κB family members such as RelA and RelB, thus influencing the expression of specific NF-κB dependent target genes. While additional work is needed to fully understand the functional role of the interaction with Rel proteins, these prototypical observations highlight how AhR biology is more complex than suggested by the classic AhR/ARNT signaling pathway.

### 3.6. AhR/CLOCK

*AHR*, *ARNT*, and *CYP1A1* genes display circadian variation and are characterized by diurnal rhythmicity, with increased expression during the day [101,102,103]. In mammals, the molecular circadian clock consists of transcriptional activators and repressors that are regulated by positive and negative feedback loops [104]. The transcriptional activators, i.e., CLOCK (circadian locomotor output cycles kaput) and BMAL1, heterodimerize through their PAS domains by binding at enhancer-box (Ebox) regions to enhance transcription of specific clock genes [105].

The classic AhR partner, ARNT, shares significant sequence similarity with BMAL1. Thus, murine AhR may also interact with BMAL1 [106]. Specifically, quantitative real-time PCR and immunoblot analyses demonstrated that TCDD exposure alters the expression of the canonical clock genes, *BMAL1* and *PER2*, in mouse ovary cells. AhR transcript and protein, which display a circadian pattern of expression in the ovaries of control mice, were also altered by TCDD treatment. Furthermore, coimmunoprecipitation experiments demonstrated the time of the day-dependent interactions of AhR with BMAL1, which were increased after TCDD treatment [106].

In another study, the effects of AhR activation on rhythmic *PER1* transcripts was also examined in livers of mice after treatment with TCDD, showing that TCDD alters the *PER1* rhythm in the mouse liver and that *PER1* gene suppression depends on the presence of AhR [107]. The molecular mechanisms of *PER1* repression by AhR were determined in hepatoma cells using TCDD and beta-naphthoflavone as AhR activators. In particular, AhR interaction with BMAL1 attenuated CLOCK-BMAL1 activity and decreased CLOCK binding at Ebox1 and Ebox3 in the *PER1* promoter, thus delaying the *PER1* rhythm [107]. Overall, although an interaction between circadian factors and AhR has been reported, how AhR activation would impact physiological rhythmicity remains to be determined.

## 4. Coactivator and Corepressor Families as Ancillary Proteins in AhR-Dependent Gene Transcription

The direction of gene transcription, i.e., induction or repression, is not determined by the transcription factor itself but by the specific recruitment of either coactivators, i.e., nuclear receptor coactivators (NCOAs), or corepressors (NCORs) to the transcriptional complex [108]. The intrinsic functions of transcription factors are therefore modulated by the recruitment of these ancillary proteins, which favor the interaction with histone acetyl transferase (HAT; for NCOAs) or histone deacetylase (HDAC; for NCORs) enzymes that promote or inhibit gene transcription, respectively [109,110]. However, because there is evidence that an NCOA can act as NCOR and vice versa depending on specific contexts [111], the term ‘coregulator’ is now considered more appropriate to identify both NCOAs and NCORs.

Coregulators lack a DNA binding domain and therefore require a direct (true coregulators) or indirect (co-coregulators) interaction with transcription factors—either ligand or not ligand activated (as AhR)—to be recruited as enhancers of promoters of target genes [112]. In fact, such complexes often contain several individual proteins and their composition can vary in a highly dynamic manner [113]. Since the discovery of the first coregulator, i.e., steroid receptor coactivator 1 (SRC-1; now known as NCOA1), in 1995 [114], more than 450 coregulators and hundreds of co-coregulators have been identified. Notably, coregulators are versatile proteins that influence not only transcriptional initiation but also elongation, splicing, and translation. In fact, coregulators are endowed with sensing properties that integrate the cellular environment and signaling pathways to the transcriptional output [115]. Accumulating evidence points to the pivotal roles that coregulators play in inflammatory and metabolic pathways, both under homeostasis and in disease [116], in which AhR is clearly involved. For this reason, they have recently become the focus of studies aimed at investigating coactivator interactions with AhR as novel drug targets [117].

### 4.1. SRC-NCOAs Family

NCOA1, NCOA2 (previously known as SRC-2), and NCOA3 (SRC-3/pCIP) belong to the p160 SRC family and have similar amino acid sequences and domain architecture. NCOA1−3 are composed of the HLH-PAS domain on the *N*-terminus, a serine/threonine-rich domain, a nuclear receptor interaction domain (NRID) in the middle, and two transactivation domains on the *C*-terminus [118,119]. The HLH-PAS domain contains the nuclear localization signal and is homologous to the DNA-binding region of HLH-containing transcription factors, including AhR and ARNT, but no evidence has been provided so far for a direct binding of DNA by NCOA1−3. Instead, the HLH-PAS domain can bind to transcription factors, other coregulators (i.e., CBP and p300), or chromatin remodelers [120]. The NRID can also bind to transcription factors. NCOAs are subjected to a variety of post-translational modifications, including phosphorylation, acetylation, methylation, ubiquitination, and SUMOylation throughout different domains and regions, which allow the integration between a myriad of signaling networks and gene transcription [121]. Knockout models for NCOA1−3 possess a range of phenotypic defects, mostly in the reproductive and metabolic functions. NCOA1 has been shown to be generally over-expressed in breast cancer. Although initially identified as NCOA, NCOA4, 5, 6, and 7 are distantly related to NCOA1−3 and have been less characterized. In particular, NCOA4 was identified in a yeast two-hybrid screen as an androgen receptor (AR) interacting protein and was shown to potentiate AR transcriptional activity [3] and, based on these findings, it was referred to as AR-associated protein 70 (ARA70) [122]. Subsequent studies, however, indicated that NCOA4 interacts with and regulates the function of additional nuclear receptors [123]. NCOA5 was reported to possess coactivator and corepressor functions, which can modulate ERα-mediated transcription [124]. NCOA6, also known as AIB3, PRIP, ASC-2, TRBP, RAP250 or NRC, is an essential coactivator for embryonic development, as knockout of the *Ncoa6* gene in mice results in embryonic lethality accompanied with defective development of the placenta, heart and liver [125]. Specific studies suggest that NCOA6 may play an important role in multiple signaling pathways regulated by hormones and growth factors [126,127]. NCOA7, also known as estrogen receptor-associated protein 140, is involved in potentiating the transcription activation of various nuclear receptors, including ERα, ER-β, peroxisome proliferator-activated receptor γ, and retinoic acid receptor-α, and mediates receptor signaling in specific target tissues [128].

The interaction of AhR with NCOA family members has been reported to modulate AhR transcriptional activity [129]. Indeed, previous studies showed that SRC-1 and NCOA-2 are capable of interacting with ARNT in vivo after transient transfection into mammalian cells [130]. Specifically, SRC-1 and NCOA-2 were found to associate with the *CYP1A1* gene enhancer region in a TCDD-dependent fashion, leading to TCDD enhanced gene transcription [130]. Additional evidence suggests that the specific ligand activating AhR may also influence this interaction. In the case of AhR interaction with agonists such as TCDD or FICZ, the ordinary transcription recruits NCOA1, NCOA2, and NCOA3 (each present on the promoter) enhancing *CYP1A1* transcription. In contrast, an AhR antagonist such as 3,3′-diindolylmethane (DIM) promotes AhR nuclear translocation and p160 coactivator recruitment but fails to recruit potential coactivator proteins and thus to activate Pol II or histone acetylation. These data suggested that ligand-dependent changes in AhR conformation can affect its transcriptional activity through the recruitment of coactivator family members at the promoter of target genes [22]. NCOA4 was found to bind and enhance AhR activation by TCDD. Interestingly, NCOA4-facilitation of AhR activity was abolished by the overexpression of androgen receptors, suggesting a potential competition of AhR and androgen receptor for NCOA4 [131]. At present, no specific interactions between AhR and NCOA5 or NCOA6 coregulators have been documented. Until recently, no ligands directly binding NCOAs and capable of promoting their interaction with nuclear receptors have been reported. However, we identified NCOA7 as the molecular target of the metabolite 3-hydroxyanthranilic acid (3-HAA), a Trp metabolite produced downstream along the IDO1-mediated kynurenine pathway. Specifically, in mouse DC subsets, the presence of 3-HAA increased the association of NCOA7 and AhR, resulting in enhancement of kynurenine-driven, AhR-dependent gene transcription [132]. These results represented the first evidence that specific endogenous molecules, such as those produced by metabolism of the essential amino acid Trp, can be actively involved in the modulation of NCOA protein activity, thus leading to the enhancement of AhR transcriptional activity. Since different cell types may express different quantities of such activators, this may represent an interesting means to modulate AhR function in specific sites or cells.

### 4.2. CBP and p300

cAMP response element-binding protein (CREB)-binding protein (CBP) and p300 are two closely homologous proteins involved in multiple biological processes. They function as coregulators and also as HATs [133,134]. In addition to modulating the transcriptional activity of several nuclear receptors, CBP and p300 act as integrators of various cellular signaling pathways, including the WNT/β-catenin signaling pathway that is involved in cell proliferation and tumor development [135]. Moreover, CBP and p300 are often involved in the signaling pathways of immunocytes but the outcome can be different in distinct cells and contexts. For example, on the one hand, they favor the expression of several cytokines (mainly via interaction with NF-κB [136]), including IFNs during viral infection (via interaction with IFN-regulatory factors or IRFs) [137]. On the other hand, they drive the differentiation of Treg cells [138].

Particularly interesting in the present context is the finding that CBP and p300 act as transcriptional activators of ARNT [139]. However, in the presence of a potent toxic xenobiotic AhR ligand such as TCDD, mouse AhR activation leads to CBP/p300 displacement and the inhibition of cell cycle signaling in tumor cells [140], suggesting a possible protective mechanism.

The ability of AhR to inhibit NF-κB has been linked to both a direct interaction between AhR and NF-κB/RelA (see Section 3) and also to a potential mutual repression by the competitive binding of ligand-AhR/ARNT complexes or RelA for p300/CBP [91]. Indeed, it is possible that the mutual modulation between AhR and NF-κB can be mediated by a transcription coactivator, such as p300/CBP. Intriguingly, p300/CBP was found to associate with both RelA [141] and ARNT [139]. Thus, competition between ligand-AhR/ARNT complexes and RelA for p300/CBP binding could affect the levels of transcriptional activation seen in these two pathways.

A recent study evaluating the rapid dynamics of the CBP/p300 acetylome indicated that CBP/p300 affects AhR signaling at multiple steps. Interestingly, it was found that CBP/p300 acetylates ARNT, which binds to AhR to promote AhR-dependent transcriptional activation [142]. Most notably, treatment with CBP/p300 acetyltransferase inhibitors completely blocked *Cyp1a1* gene induction in response to the AhR ligand FICZ in both MEF and Kasumi-1 cells. These results showed that CBP/p300 catalytic activity is indispensable for AhR-dependent *Cyp1a1* transcription [142]. Moreover, these data showed that CBP/p300 catalytic activity is required for *Cyp1a1* transcriptional maintenance, and that loss of this activity may result in nearly immediate transcriptional arrest.

### 4.3. PRMT

Protein arginine methyltransferases (PRMTs) (a family comprising at least nine members) catalyze histone arginine methylation leading to the formation of monomethylarginine as well as symmetrical and asymmetrical dimethylarginine. Therefore, PRMTs are regulating enzymes that participate in chromatin remodeling through histone post-translational modification and are thus considered as epigenetic modulators. These modifications affect the function of target proteins and regulate their ability to interact with DNA, RNA, or other proteins [143]. According to recent data, PRMT1 and PRMT4−6 are the most important in modulating inflammation and immune responses [144]. As an example, PRMT4 (also known as coactivator-associated arginine methyltransferase 1), one of the members of the PRMT family that regulates multiple cellular functions, acts as a primary coactivator by enhancing NF-κB recruitment to cognate sites and by controlling transcription in a gene-specific manner [145]. Mice with a conditional deletion of *Prmt5* in Treg cells develop severe, scurfy-like autoimmunity due to a downregulation of FoxP3, the master transcription factor of Treg cells [146].

In mice, PRMT4 acts as an NCOA in the induction of the *Cyp1a1* gene by AhR activated by TCDD. Accordingly, the treatment of HepG2 cells with silencing RNA targeting PRMT4 significantly decreased the *CYP1A1* gene expression level [147].

## 5. Conclusions

**Figure 2:**
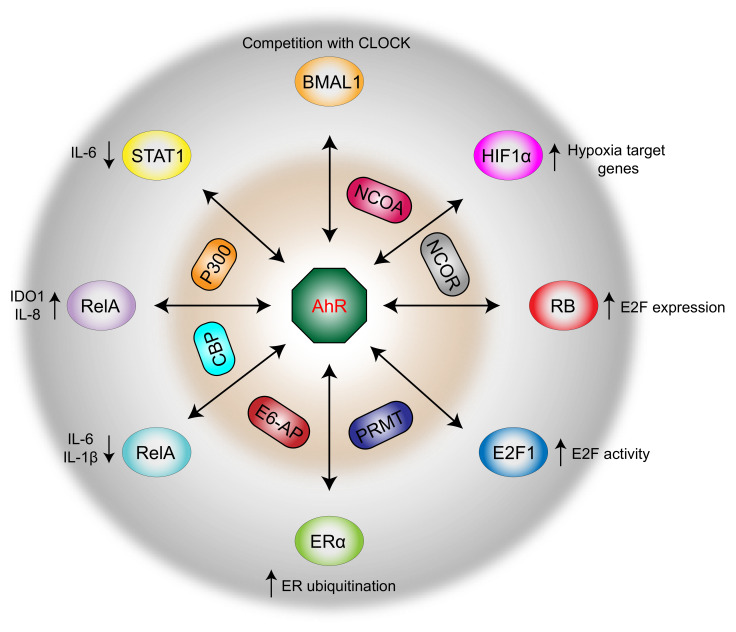
The multiple trajectories regulating immune and nonimmune functions. The modulation of AhR-dependent gene transcription is achieved through the recruitment of specific transcription factors and coregulators, either coactivators (here in red) or corepressors (here in grey). Besides canonical signaling, AhR can directly interact with ER, RB, E2F1, STAT1, NF-κB transcription factors RelA and RelB, leading to the modulation of estrogenic signals, cell cycle progression, apoptosis, and inflammatory responses, respectively. By competing with AhR, HIF1α binds the nuclear translocator ARNT and thus enhances the expression of hypoxia target genes and indirectly suppresses the AhR activity. AhR, by heterodimerizing with the transcriptional activator BMAL1, interferes with the transcription of CLOCK target genes. Specific coactivators (including NCOA family members and PRMT) drive AhR activity directly or indirectly (via CBP and p300) by promoting the transcription of the nuclear translocator ARNT. BMAL1, brain and muscle ARNT-Like 1; CBP, cAMP response element-binding protein (CREB)-binding protein; CLOCK, circadian locomotor output cycles kaput; E6-AP, ubiquitin E3-ligase; E2F1, E2F Transcription Factor 1; ERα, estrogen receptor; HIF1a, hypoxia-inducible factors; NCOA, nuclear receptor coactivators; NCOR, nuclear receptor corepressors; PRMT, protein arginine methyltransferases.

Dysregulation of coregulator signaling circuitry has severe consequences for cell homeostasis and often contributes to pathogenesis. Much research on transcription factor biology and their genetic pathways has been undertaken over the last 30 years, yet very little is known about the molecular modalities of dynamic interactions between specific transcription factors and other protein partners, genomic DNA, and coregulators. The current knowledge about the array of transcription factors and coregulators would indicate that, by interacting with AhR, they tune its transcriptional activity in response to endogenous and exogenous ligands (Table 1). The ligand-activated transcription factor AhR mediates a great array of functions, especially in the regulation of immune responses [7]. Currently known AhR regulatory networks (Figure 2) are likely to be a small representation of all the interactions that may occur in vivo. Even in yeast, where this aspect of AhR has been explored under standard laboratory conditions [148,149], the information on regulatory pathways is likely far from being complete. Therefore, novel studies will need to examine and further explore the relevance of the landscape of such AhR interactions in vivo.

From a pharmacological viewpoint, protein–protein interactions are likely to represent valuable alternative classes of therapeutic targets. As described above, AhR mostly acts via protein–protein interaction, a process further modulated by endogenous and exogenous molecules [150]. Acting on this level may well be at the forefront of drug development. The current view of transcription regulation by AhR would suggest that tackling AhR interaction with specific partners or overexpression of coactivators and/or downregulation of corepressors could modify AhR-dependent outcomes in specific sites in response to specific ligands. As more attempts at modulating transcription factor activity are undertaken, and structural data are acquired and useful information is integrated into rational drug design, valuable knowledge will be gained on specific strategies for developing drugs targeting transcription factors. Key discoveries will spawn into new therapeutic approaches not limited to AhR but also to similar classes of transcription factor molecules.

## Figures and Tables

**Table 1:** The landscape of factors regulating AhR functions.

Regulator Name	Acronym	Function When Interacting with AhR or ARNT
**Transcription Factors**		
Hypoxia-inducible factor	HIF1 α	Enhanced expression of hypoxia target genes and indirect suppression of AhR activity
Retinoblastoma	RB	Repression of E2F-dependent gene expression. Protection of RB from CDK2-mediated phosphorylation
-	E2F1	Activation or repression E2F1 target genes in different target cells
Estrogen receptor α	ERα	Modulation of ERα target genes
Androgen receptor	AR	Repression of AR transcriptional activity
Nuclear factor kappa-light-chain-enhancer of activated B cells	NF-κB	Suppression of inflammatory immune responses
Circadian locomotor output cycles kaput	CLOCK/BMAL1	Enhanced transcription of specific clock genes.
**Coregulators**		
Nuclear receptor coactivators	SRC-NCOAs family	Modulation of AhR transcriptional activity by recruitment of NCOA1, NCOA2, NCOA3, NCOA4 and NCOA7.
CREB-binding protein	CBP	Transcriptional activator of ARNT.
	P300	CBP/p300 activation for AhR-dependent Cyp1a1 transcription
Protein arginine methyltransferases	PRMTs	Involvement of PRMT4 in Cyp1a1 gene induction by TCDD.

## Data Availability

Not applicable.

## Abbreviations

3-HAA	3-hydroxyanthranilic acid
3-MC	3-methylcholanthrene
AhR	aryl-hydrocarbon receptor
APC	antigen presenting cells
AR	Androgen receptor
DCs	dendritic cells
DRE	dioxin responsive element
EAE	experimental autoimmune encephalomyelitis
ER	estrogen receptor
ERE	estrogen responsive elements
FICZ	6-formylindolo[3,2-b]carbazole
HAT	histone acetyl transferase
HDAC	histone deacetylase
HLH	helix–loop–helix
HSP	heat shock protein
I3C	indole-3-carbinol
Iald	indole-3-aldehyde
ID	interaction domain
IO	indirubin-3′-oxime
KC	keratinocyte chemoattractant
Kyn	kynurenine
LPS	lipopolysaccharide
NCOA	nuclear receptor coactivator
NCOR	nuclear receptor corepressor
PRMTs	protein arginine methyltransferases
RB	retinoblastoma
RD	repression domain
siRNA	small interfering RNA
TCDD	2,3,7,8-tetrachlorodibenzo-p-dioxin
Tf	transcription factor
Th	helper T lymphocyte
TLR	Toll like receptor
Tr1	regulatory T cells type 1
Trp	tryptophan
XRE	xenobiotic responsive element
β-NF	β-naphthoflavone
